# The Synergistic Anti-Apoptosis Effects of Amniotic Epithelial Stem Cell Conditioned Medium and Ponesimod on the Oligodendrocyte Cells

**DOI:** 10.3389/fphar.2021.691099

**Published:** 2021-06-21

**Authors:** Fahimeh Safaeinejad, Sareh Asadi, Shiva Ghafghazi, Hassan Niknejad

**Affiliations:** ^1^ Department of Pharmacology, School of Medicine, Shahid Beheshti University of Medical Sciences, Tehran, Iran; ^2^ Neurobiology Research Center, Shahid Beheshti University of Medical Sciences, Tehran, Iran

**Keywords:** amniotic membrane, stem cells, multiple sclerosis, cuprizone, ponesimod

## Abstract

Multiple sclerosis is a chronic inflammatory and neurodegenerative disease of the central nervous system. The current treatment of Multiple sclerosis is based on anti-inflammatory disease-modifying treatments, which can not regenerate myelin and eventually neurons. So, we need new approaches for axonal protection and remyelination. Amniotic epithelial stem cells amniotic epithelial cells, as a neuroprotective and neurogenic agent, are a proper source in tissue engineering and regenerative medicine. Due to differentiation capability and secretion of growth factors, AECs can be a candidate for the treatment of MS. Moreover, sphingosine-1-phosphate (S1P) receptor modulators were recently approved by FDA for MS. Ponesimod is an S1P receptor-1 modulator that acts selectively as an anti-inflammatory agent and provides a suitable microenvironment for the function of the other neuroprotective agents. In this study, due to the characteristics of AECs, they are considered a treatment option in MS. The conditioned medium of AECs concurrently with ponesimod was used to evaluate the viability of the oligodendrocyte cell line after induction of cell death by cuprizone. Cell viability after treatment by conditioned medium and ponesimod was increased compared to untreated groups. Also, the results showed that combination therapy with CM and ponesimod had a synergistic anti-apoptotic effect on oligodendrocyte cells. The combination treatment with CM and ponesimod reduced the expression of caspase-3, caspase-8, Bax, and Annexin V proteins and increased the relative BCL-2/Bax ratio, indicating inhibition of apoptosis as a possible mechanism of action. Based on these promising results, combination therapy with amniotic stem cells and ponesimode could be a proper alternative for multiple sclerosis treatment.

## Introduction

Multiple sclerosis (MS) is a neurodegenerative and inflammatory disease that affects both the central nervous system (CNS) and the immune system (Esposito et al., 2018). MS is the second most common cause of non-traumatic neurological disability among young adults (Vargas and Tyor, 2017). The hallmark of MS is immune-mediated demyelination and axonal degeneration which leads to myelin sheath deficiency and subsequent neurological dysfunctions (Podbielska et al., 2013). Today, the management of MS is based on disease-modifying therapeutics (DMTs) which decrease relapse and disability by reducing inflammation; but their long-term benefits stay indeterminate. These drugs include interferons, glatiramer acetate (GA), and monoclonal antibodies (mAb) such as natalizumab. All of the DMTs are injections that reduce patient compliance (Hauser and Oksenberg, 2006; Safaeinejad et al., 2018). To overcome this problem, the first oral drug was approved by the FDA in 2010. Sphingosine-1-phosphate (S1P receptor modulators are the new therapeutic agents for MS that are orally administered. The prototype of these drugs is fingolimod that regulates five subtypes of S1P receptors (Brossard et al., 2014). The results of clinical trials have shown that fingolimod effectively reduces the number of relapses, new plaques on MRI, and the progression of disability, and prevents the reduction of brain volume. Unfortunately, this drug causes cardiovascular complications due to its nonspecific effects on sphingosine 1-phosphate (S1P) receptors (with an off-target effect on S1P3). Another drug of this group is ponesimod which acts more selective and specific (Chaudhry et al., 2017; Chun et al., 2021). It targets the protein S1P1 and traps immune cells in lymph nodes, which prevents them from doing damage to the CNS. It has been shown that this drug can significantly reduce plaque volume as well as myelin and axon destruction in the brain, spinal cord, and cerebellum in the mouse model of MS (You et al., 2013). Ponesimod can cross the blood-brain barrier and attach to neurons, oligodendrocyte progenitor cells, astrocytes, and microglia in the CNS by binding to its receptor (D’Ambrosio et al., 2016).

Despite the palliative and preventive effects of these drugs, there is no cure for MS and patients suffer a chronic and progressive disability. Thus, effective regenerative treatment with fewer side effects is needed. New therapies must have three main characteristics containing an immunomodulatory effect in the CNS to inhibit the activity of inflammatory cells and their mediators, a neuroprotective role to prevent the destruction of remaining healthy neurons, and finally, neurogenesis capability to produce neural cells (especially oligodendrocytes) to replace damaged cells with new and functional cells (Zhornitsky et al., 2013).

From the regenerative point of view, stem cell therapy is a promising approach to treat multiple sclerosis. The first attempts to use stem cell therapy for MS have been done more than 15 years ago. It has been shown that myelin degradation has decreased using stem cells in an animal model of MS (Rahim et al., 2018). A variety of stem cells has been employed in MS-related cell therapies. Their positive features such as immunomodulatory and anti-inflammatory effects (El-Akabawy and Rashed, 2015), neural differentiation (Joggerst and Hatzopoulos 2009), and neuroprotective and neurotrophic properties in animal models (Connick et al., 2012) make them a suitable candidate for the regeneration of neural damage in MS (Soleimani et al., 2016; Barati et al., 2020). Among the stem cells, amniotic epithelial cells (AECs) are a promising source for cell transplantation in the treatment of MS due to their specific characteristics. Low immunogenicity, lack of ethical considerations, extraction of a large number of cells from a placenta, and secretion of growth factors are some characteristics that have largely circumvented the challenges of stem cells (Lavallard et al., 2018). These cells are pluripotent and can differentiate into three embryonic cells: ectoderm (neuronal and glial cells), mesoderm (heart cells), and endoderm (pancreas and liver). They also have self-renewal property without being tumorigenic. hAECs express immunomodulatory factors such as IL-10, TGF-β, IDO, HGF, prostaglandin E2, and HLA-G. They also do not express HLA-B (CLASS I) and HLA-DR (CLASS II), which result in the reduced possibility of immunological reactions and cell transplant rejection. Moreover, these cells have anti-inflammatory and antimicrobial properties) AECs express growth and angiogenic factors EGF, GRO, VEGF, TIMP-1, PDGF, IGF-1, G-CSF, and GM-CSF. AECs release several neurotrophic factors such as brain-derived neurotrophic factor (BDNF), and neurotrophin (NT3) (Niknejad et al., 2008; Miki et al., 2005; Zhang et al., 2015) which play an important role in stimulating the growth and direction of neural cells (Wang et al., 2013). Also, recent studies revealed that hAECs release anti-apoptotic factors include GDF5/9/11, TGF-β1/2/3, and BMP15 (Zhang et al., 2017). These characteristics suggest AECs as an appropriate choice in cell therapy of MS.

Because the pathogenesis of MS is complex, it is assumed that combination therapy of hAECs conditioned medium (CM) and ponesimod can be effective in the treatment of MS. This study aimed to evaluate the effects of AECs derived CM and ponesimod and their possible synergistic effects on the oligodendrocyte cell viability after induction of apoptosis by a toxic cuprizone model to investigate the direct influence of cuprizone on cell viability *in vitro*. Moreover, the expression of caspase three and caspase 8, Bax, and Annexin V (as apoptotic markers), and the expression of BCL-2 were assessed following treatment of oligodendrocyte cells with hAECs-CM and ponesimod. In this study, we used cuprizone for induction of apoptosis in oligodendrocytes. Cuprizone is a toxic agent which induces apoptosis in the oligodendrocytes *in vitro* and *in vivo*. It is generally used as an inducer of multiple sclerosis (Rodnichenko and Labunets, 2018).

## Materials and Methods

### Isolation of AECs and Conditioned Medium Collection

All experimental procedures were done following the guideline for laboratory research after approval by the ethics committee of Shahid Beheshti University of Medical Sciences.

The human placenta was received after elective cesarean delivery from Erfan hospital. The informed consent was received from the parents. Isolation of AECs was done as described in our previous study (Biniazan et al., 2020). Briefly, the amnion layer of the human placenta was mechanically peeled off from the chorion, and incubated by the 0.15% trypsin-EDTA at 37°C for 10 min. Then the supernatant was discarded to exclude debris. AECs were isolated from the second and third 40-min digests. Then AECs suspended in DMED/F12 containing 100 U/ml penicillin/streptomycin solution and 10% heat-inactivated FBS and cultured in T25 flasks at a density of 2×10^4^ cells per cm^2^ for 5 days. After reaching confluency (80%), the culture medium was removed and the cells were washed twice with phosphate-buffered saline and incubated again with only 15 ml DMED (without serum and antibiotic). After an additional 24 h, the replaced medium was separated from the cells. The collected supernatant filtered through a 0.22-μm filter to remove all the possible epithelial cells from the medium.

### Ponesimod Preparation

Ponesimod [(*Z*, *Z*)-5-[3-chloro-4-((2*R*)-2,3-dihydroxypropoxy)-benzylidene]-2-propylimino-3-o-tolyl-thiazolidin-4-one] (Adooq Bioscience, United States of America) was dissolved in DMSO and then diluted to the suitable concentrations. Also, different concentrations of ponesimod (0, 2.85, 5.7, 10, 100, and 1,000 nM) on untreated oligodendrocytes were investigated.

### Cuprizone Treatment

The stock solution (1 ml) of the cuprizone was provided freshly. For this purpose, cuprizone [bis(cyclohexanone)oxaldihydrazone] powder (Sigma-Aldrich, United States) was dissolved in 50% ethanol at 37°C under stirring 250 rpm for 20 min to obtain the concentration of 1 mM (8). Then this stock was diluted to reach the concentrations 0, 25, 50, 75, 100, 125, and 150 μM of cuprizone. To check the toxicity of the vehicle, the same concentrations of 50% ethanol (0, 25, 50, 75, 100, 125, and 150 μM) were used.

### Cell Culture and Cytotoxicity Assay

The cytotoxic effect of cuprizone was evaluated using OLN-93, as an oligodendroglial cell line (Pasteur Institute, Iran). These cryopreserved cells were defrosted and resuspended in Dulbecco’s modified Eagle’s medium (DMEM) (Gibco, United Kingdom), 10% heat-inactivated fetal bovine serum (Gibco, United Kingdom), and 100 U/ml penicillin-streptomycin (Thermo Fisher, United States) and cultured in flasks at 37°C and atmosphere of humidified air enhanced with 5% CO_2_. After reaching 75% confluence, the cells were detached by 0.15% Trypsin/EDTA enzyme and seeded in the 24-well plates with a density of 2.5 ×10^5^ cells per cm^2^ for 24 h in 5% CO_2_ at 37°C overnight. To evaluate the cytotoxic effect of each sample, the OLN-93 cells were treated with different concentrations (0, 25, 50, 75, 100, 125, and 150 µM) of cuprizone for 24 and 48 h at 37 °C in 5% CO_2_. The cells treated with alone DMEM were considered as control. The medium in the well without cells was used as blank. As described above, the same concentration of ethanol 50% was used as a vehicle group. The IC_50_ (half maximal inhibitory concentration) of cuprizone was estimated in this step. After the OLN-93 cell line was exposed to the IC_50_ concentration of cuprizone for 24 h, they were treated with different concentrations of CM of AECs (0, 2.5, 5, 10, 25, 50, 100,150 µl), ponesimod in 5.7 nM (EC_50_), or both of them. Also, the concentration of CM to increase the proliferation of OLN-93 by 50% was determined as half-maximum effective concentration (EC_50_) of hAECs- CM.

Cell survival was examined using MTT assay (3-(4,5-dimethylthiazol-2-yl)-2,5-diphenyl tetrazolium bromide) (Sigma-Aldrich, United States of America), as we described previously ^22^. In brief, for obtaining a stock solution, 5 mg of MTT was dissolved in 1 ml PBS and filtered. 40 µl of MTT was added into each well of a 24-well plate and plate incubated in 5% CO2 at 37°C for 4 h. After that, MTT was discarded and after adding 900 µl DMSO per well, the absorbance of dissolved formazan was evaluated at 570 nm by spectrophotometer (BioTek, United States). The viability rate was determined via the following equation:

$$\mathrm{Viability}\;\text{Percentage=}\frac{\left[\mathrm{abs}\right]\text{treatment−}\left[\mathrm{abs}\right]\mathrm{blank}}{\left[\mathrm{abs}\right]\text{control−}\left[\mathrm{abs}\right]\mathrm{blank}}\times 100$$

Moreover, to determine the effect of CM on the proliferation of untreated oligodendrocyte cell line, 2.5×10^4^ OLN-93 cells were cultured in the 24-well plates at 37°C with 5% CO_2_. Then, oligodendrocyte cells (without pretreatment with cuprizone) were incubated with different concentrations of CM (0, 2.5, 5, 10, 25, 50, 100, and 150 μls) for 7 days. Finally, the proliferation of oligodendrocytes was examined by MTT assay.

### Apoptosis Assay by Flow Cytometry

Flow cytometric assay of apoptosis was performed via the Phosphatidyl Serine Detection kit (Annexin V FITC, IQ products) according to the manufacturer's protocol. The early phase of apoptosis is detected by Annexin V positive cells, while uptake of propidium iodide (PI) is an indicator of necrosis. Flow cytometry was performed by a fluorescence-activated cell sorter (BD FACS Calibur; BD biosciences, San Jose, CA, United States of America), and data were analyzed by FlowJo software.

### Western Blotting

After the treatment of the OLN-93 cells with CM and ponesimod for 24 h, total proteins were extracted from the OLN-93 cell line with lysis RIPA buffer (MgCl2 1.5 mM, HEPES 20 mM, EGTA 5 mM, EDTA 2 mM, dithiothreitol 0.1 mM, phenylmethylsulfonyl fluoride 0.1 mM, pH 7.5) (Santa Cruz Biotechnology) and spun down 12,000 rpm for 20 min. The concentration of protein was measured by the Bradford assay (detergent compatible Bradford assay kit, Thermo Scientific). Then cell lysates were diluted in RIPA buffer (NaCl (1 M), Sodium deoxycholate (0.5%), 1 mM sodium orthovanadate, Nonidet P-40 (1%),1 mMNaf, protease Inhibitors tablet (Roche), Tris (50 mM, pH 7.4), SDS (0.1%), ddH2O) to the gel-loading concentration of proteins (2.5 μg/μl) and mixed with equal volumes of sample buffer. The proteins of samples were separated using the electrophoresis process on SDS-PAGE gels. Then proteins were blotted on polyvinylidene fluoride membranes (PVDF) for 3 h by a blotting apparatus. The membrane was blocked with 5% BSA in Tris-buffered saline for 25 min and then incubated at 4°C with primary antibodies overnight: anti-Bax (1:1,000, cat.No #2772, Cell Signaling), anti-BCL-2 (1:1,000, cat.No #2876, Cell Signaling), anti-Caspase-3 (1:2,000, cat.No 235412, Merck Millipore), anti-Caspase-8 (1:1,000, cat.No #9496, Cell Signaling) and anti-GAPDH (1:1,000, cat.No #5174, Cell Signaling). The membranes were washed 3 times (5 min each time) after the incubation with buffer containing 0.1% Tween-20 and incubated with the secondary horseradish peroxidase-conjugated (HRP) goat anti-rabbit IgG (1:1,000, cat.No #7074s, Cell Signaling) for 2 h at room temperature. The membranes were consequently washed and immunoreactive bands were visualized using the chemiluminescent substrate (ECL). The intensity of protein bands was measured by digital densitometry ImageJ software (National Institutes of Health, Bethesda, MD, United States). The GADPH was used as an internal standard.

### Statistical Analysis

All experiments were repeated at least triplicates and performed independently four times to confirm reproducible results. Data are presented as the mean ± SEM. The statistical analysis was done by Graph Pad Prism software. The results of treated groups were compared by analysis of variance (ANOVA) followed by Tukey’s test. *p*-value <0.05 was considered a significant difference.

## Results

### Inhibitory Effects of Cuprizone on the Viability of OLN-93 Cells

**FIGURE 1:**
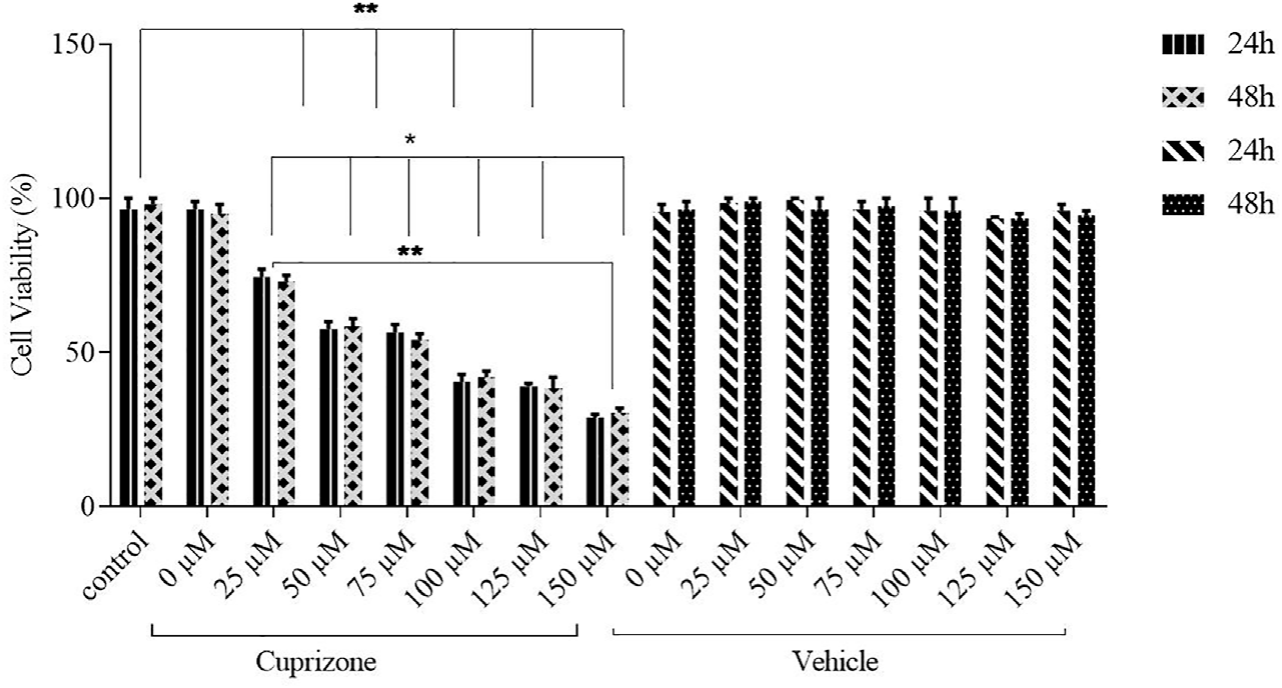
Viability of OLN-93 cells treated with cuprizone at 0, 25, 50, 75, 100, 125, and 150 µM concentrations for 24 and 48 h. As shown in the graph, cuprizone decreased the viability of oligodendrocyte cells in a concentration-dependent manner (*p < 0.05, and **p < 0.01).

**FIGURE 2:**
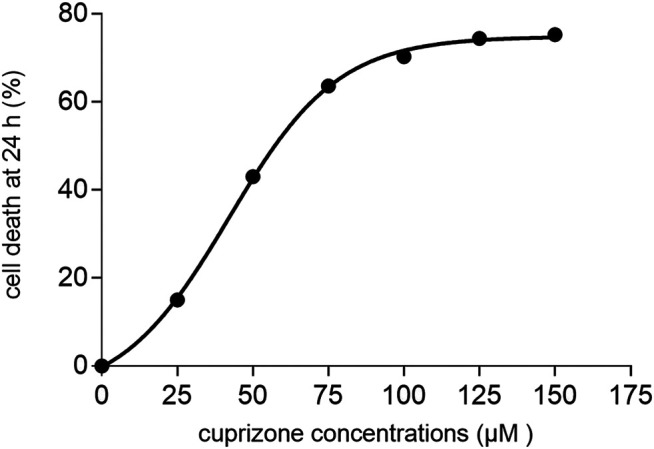
A diagram that shows the IC_50_ of cuprizone for induction of cell death in the oligodendrocyte cell line after 24 h.

Cytotoxicity assay was performed to determine the *in vitro* cytotoxicity of cuprizone. Different cuprizone concentrations (0, 25, 50, 75, 100 µM) were evaluated in 24 and 48 h on the OLN-93 cell line using MTT assay. The results revealed that cuprizone decreases cell viability dose-dependently, but we observed no differences between 24 and 48 h (Figure 1). The vehicle was not toxic in any concentration. As shown in Figure 2, the IC_50_ of cuprizone for OLN-93 cells was determined which was 49.89 µM.

#### The Effect of hAECs-CM and Ponesimod on OLN-93 Viability

**FIGURE 3:**
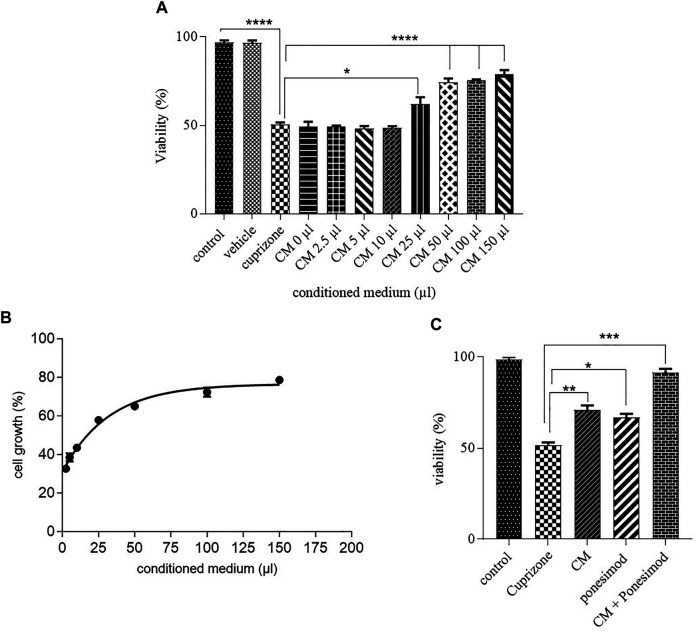
**(A)** Inhibition of oligodendrocyte cell death by hAECs-CM after 24 h (*p < 0.05, ****p < 0.0001). **(B)** a diagram that shows the EC_50_ of AECs-CM for cell viability in the Oligodendrocyte cell line after exposure with cuprizone for 24 h. **(C)** Investigation of CM and ponesimod (at concentration of EC_50_ (5.7 nM)) on oligodendrocytes survival (*p < 0.05, **p < 0.01, ***p < 0.001).

After induction of cell death by cuprizone, the effect of AECs-CM in different concentrations (0, 2.5, 5, 10, 25, 50, 100, and 150 μls) was investigated on the OLN-93 cells for 24 h. The CM inhibited *in vitro* cell death of OLN-93 in a dose-dependent manner (Figure 3A). According to the results, EC_50_ of CM was estimated after treating the oligodendrocyte cells with IC_50_ of cuprizone, which was equal to 30.78 µl (Figure 3B). The vehicle had no toxic effects on oligodendrocyte survival. To evaluate the synergistic effects of the CM and ponesimode, the EC_50_ of CM and Ponesimod (in a concentration of 5.7 nM (EC_50_)) were used simultaneously. The combination treatment of the OLN-93 cells with CM + Ponesimod increased the viability of the cells up to 91% (Figure 3C).

#### The Effect of Ponesimod on the Oligodendrocyte Cells

**FIGURE 4:**
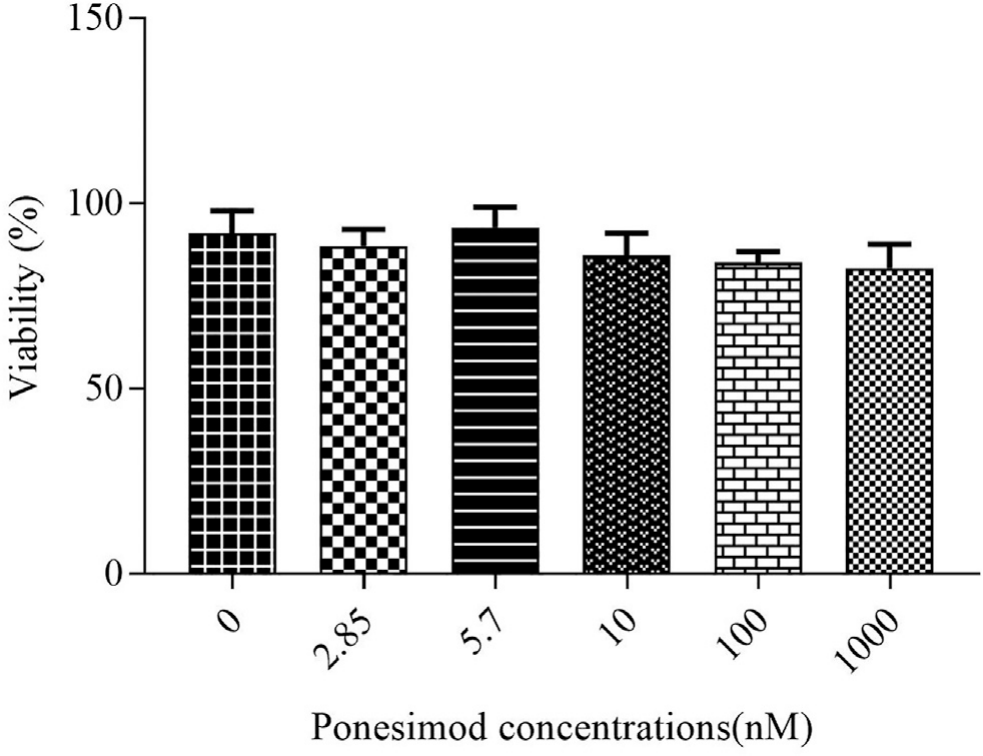
After treatment of the oligodendrocyte cell line with the ponesimod, cell survival was equal with control in all concentrations.

To investigate the effects of ponesimod on the OLN-93 cell line survival, oligodendrocyte cells (without cuprizone treatment) were treated with Ponesimod in different concentrations (0, 2,85, 5.7, 10, 100, 1,000 nM). The results showed that the serial concentrations of ponesimod had no significant effects on the viability of OLN-93 cells (Figure 4).

#### The Effect of CM on the Proliferation of the Cells

**FIGURE 5:**
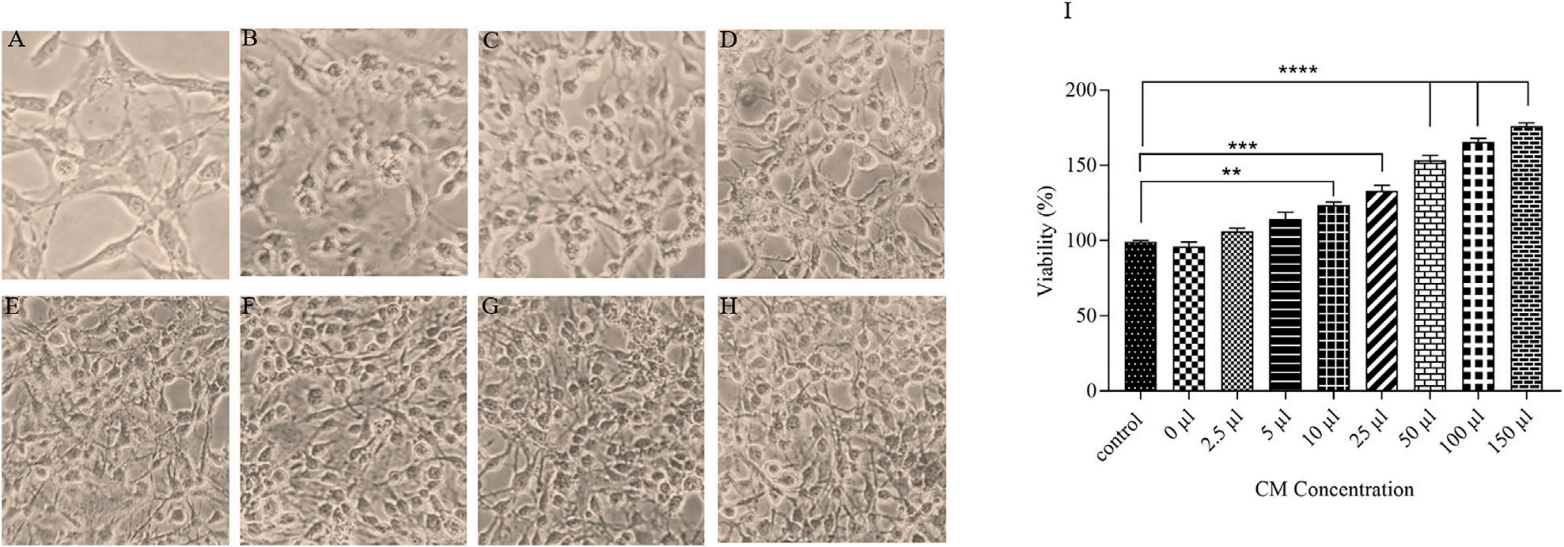
A microscopic image of OLN-93 groups treated with different concentrations of CM **(A)**: Control, **(B)**:2.5 µl, **(C)**:5 μl, **(D)**:10 μl, **(E)**:25 μl, **(F)**:50 μl, **(G)**:100 μl, and **(H)**:150 µl. **(I)** After treatment of oligodendrocytes with the CM for 7 days, cell survival was increased in all evaluated concentrations (**p < 0.01, ***p < 0.001 and ****p < 0.0001).

To evaluate the effects of ACEs condition medium on the viability of OLN-93 cell line, oligodendrocyte cells without any treatment are exposed CM for 7 days. All concentrations increased proliferation and the viability of the oligodendrocyte cells (Figures 5A–H). 150 µl of CM increased OLN-93 cell viability up to 177% (Figure 5I).

### Effect of hAECs-CM and Ponesimod on the Expression of Annexin V

**FIGURE 6:**
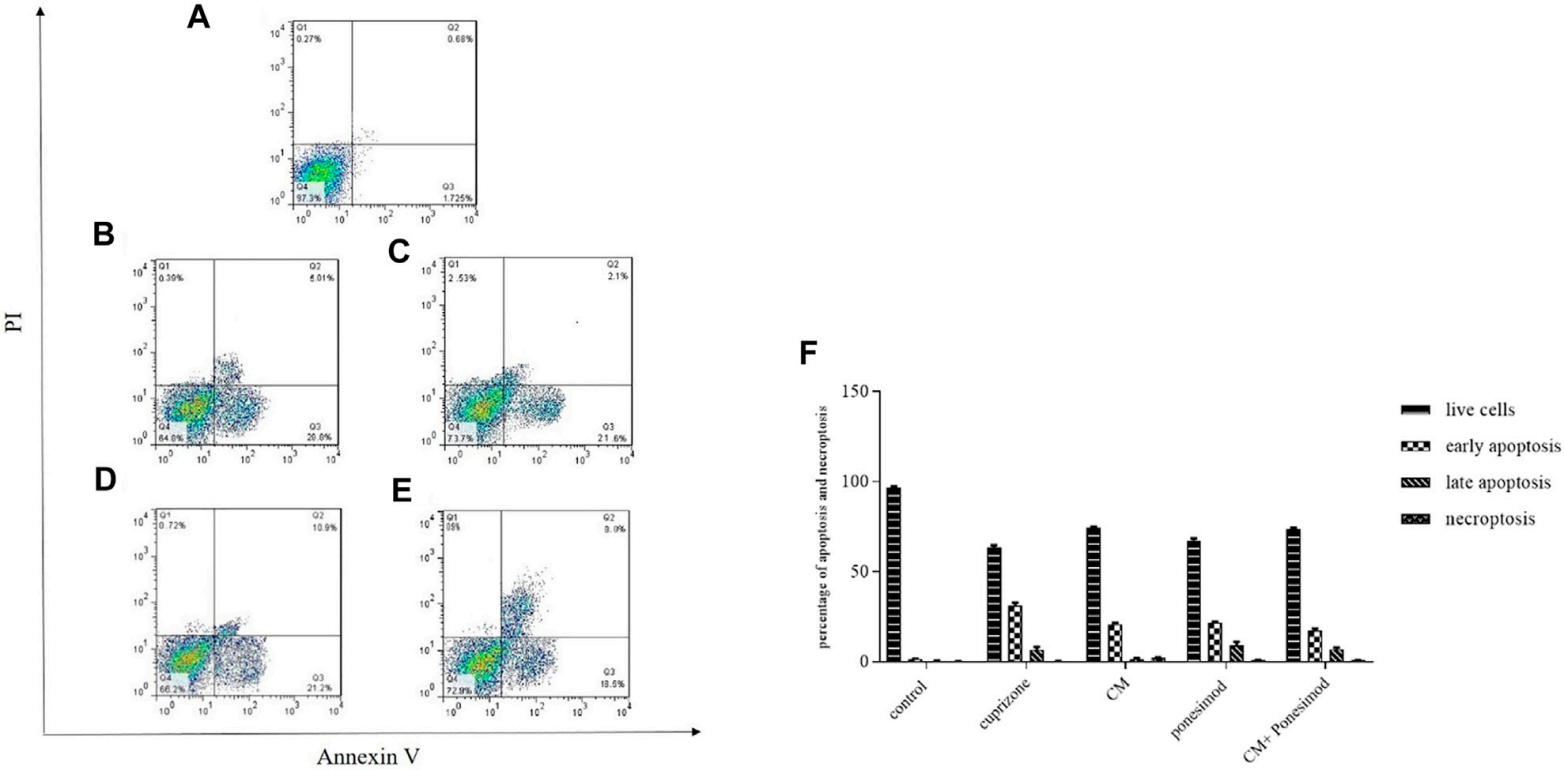
Flow cytometry analysis of annexin-V surface Annexin V to determine apoptotic cells **(A)**: control, **(B)**: cuprizone, **(C)**: CM, **(D)**: ponesimod, **(E)**: CM + ponesimod where Q1: PI^+^/Annexin V^−^ indicates necroptosis; Q2: PI^+^/Annexin V^+^ shows late apoptosis and Q3: PI^−^/Annexin V^+^ indicates early apoptosis, Q4: live cells. **(F)**: The Flow cytometry analysis graph that shows the percentages of live cells, early apoptosis, late apoptosis, and necroptosis.

The main property of apoptosis is the incoherence of the plasma membrane. During the early phase of apoptosis, Phosphatidylserine (PS) proteins translocate from the inside to the outside of the membrane that was detected by Annexin V, in the presence of Ca^2+^ ions. To diagnose apoptotic from necrotic procedures, propidium iodide (PI) was used that specified cell necrosis. The results demonstrated that cuprizone significantly increased the number of apoptotic cells (29.9%) whilst treatment of oligodendrocytes with CM and ponesimod reduced the apoptosis process as showed in Figure 6.

### Effect of hAECs-CM and Ponesimod on the Expression of Caspase-3, Caspase-8, BCL2, and Bax

**FIGURE 7:**
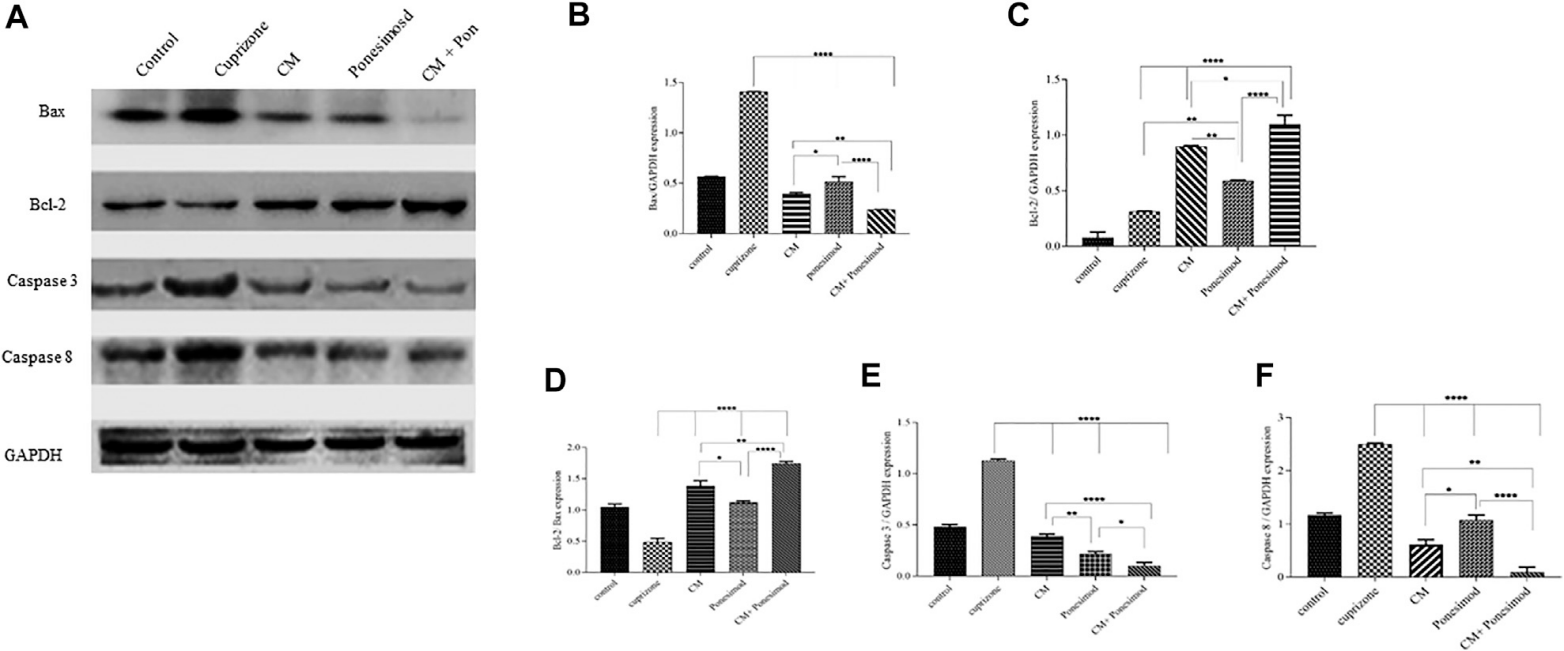
**(A)** The representative Western blot analysis of the expression of caspase3, caspase 8, BCL-2, and Bax in OLN-93 cells after treatment with CM (30.78 µl), Ponesimod (5.7 nM), and CM + Ponesimod. GAPDH was used as an internal reference **(B)** Bax/GAPDH expression **(C)** BCL-2/GAPDH expression **(D)** A histogram that shows the relative BCL-2/Bax ratio. **(E, F)** Detection of expressions of Caspases three and eight oligodendrocyte cell line after treatment with CM, Ponesimod, and CM + Ponesimod. GAPDH was used as standard housekeeping marker (*p < 0.05, **p < 0.01 and ****p < 0.0001).

To further explore the induction of apoptosis as a mechanism of action of cuprizone on OLN-93 cells, the expressions of apoptotic markers were evaluated by Western blot analysis. OLN-93 cell line incubated with cuprizone for 24 h. Then, CM and ponesimod were added to the cells about 24 h, and finally, the expression of Bax, BCL-2, Caspase3, Caspase8, and GAPDH (as an internal standard) were measured. Results revealed that the apoptotic proteins such as Caspase3, and Caspase8 in the cuprizone group were significantly increased while CM and Ponesimod reduced these factors (Figure 7).

The assessment of pro-apoptotic protein Bax and anti-apoptotic protein BCL-2 revealed that cuprizone causes a reduction in BCL-2 expression, but up-regulated the expression of Bax. AECs-CM and ponesimod meaningfully increased BCL-2 expression and reduced Bax expression. Moreover, treatment with AECs-CM and ponesimod increased significantly the relative BCL-2/Bax ratio in comparison to the control group (Figure 7).

## Discussion

In this study, we evaluated the effect of hAECs-CM and ponesimod, an S1P1 receptor regulator, on oligodendroglial cell line viability after induction of cell death by cuprizone. While it is not truly understood how cuprizone is toxic to oligodendrocytes, it has been hypothesized that it may be related to disruption of energy metabolism in oligodendrocytes via their mitochondria which leads to apoptosis (Yamate-Morgan et al., 2019). Cuprizone is a copper-chelating substance that induces changes in the activity of the copper-containing mitochondrial enzymes such as the Cu/Zn superoxide dismutase and cytochrome c oxidase (Acs et al., 2013; Rodnichenko and Labunets, 2018). Cuprizone reduces activities of both Complex IV and SOD that are located in mitochondria. Thus, the number of free radicals of reactive oxygen species (ROS) increases in the cells, which leads to the opening of mitochondria pores via cytochrome C that activate cytoplasmic proteolytic proteins (caspases) released from mitochondria (Redza-Dutordoir and Averill-Bates, 2016). The released proteins play a notable role in the development of apoptosis. Our result revealed that cuprizone induced apoptosis in oligodendrocyte cells via increasing Bax, capase3, and 8. This result was consistent with the study of Be´nardais et al. which showed that cuprizone causes oligodendrocyte cell death through induction of apoptosis (Bénardais et al., 2013).

To evaluate the effect of condition medium on cuprizone-induced death of oligodendrocyte cell line, different concentrations of CM were assayed. The results of the MTT assay showed that treatment of cultured OLN- 93 with hAECs-CM, inhibited dose-dependently cell death. Studies displayed that hAEC-conditioned media include many soluble factors with a variety of important biological effects (Uchida et al., 2000). hAECs secret some growth factors like basic fibroblastic growth factor (bFGF) and epithelial growth factor (EGF) which have a critical role in the survival of neural cells (Zhang et al., 2017). Moreover, they produce and release several neurotrophic factors, such as brain-derived neurotrophic factor (BDNF), neurotrophin-3 (NT-3), and nerve growth factor (NGF). BDNF is a neuroprotective factor that increases axon protection in autoimmune demyelination (Uchida et al., 2000; Yang et al., 2013). NGF and BDNF have a remarkable effect on neuronal survival. It has been shown that these growth factors act directly on neurons and prevent apoptosis. NGF and BDNF bind to their receptor TrkB and activate two signaling pathways containing the phosphatidylinositol 3-kinase (PI3K)/Akt, which leads to the deactivation of proapoptotic targets, and the extracellular signal-regulated kinase (ERK), which results in inducing transcription of different neuronal survival-related genes (Jain et al., 2013).

Moreover, the ponesimod was tested on the OLN-93 cells death. The data illustrated that ponesimod increased the survival of oligodendrocyte cells. It has been shown that the S1PR modulator administration enhances survival signaling by increasing BCL2, Sphingosine kinase 1(SphK1), Sphingosine kinase 2(SphK2), and ceramide kinase (CerK) gene expression in neural cells (Angelopoulou and Piperi, 2019). Moreover, S1PR regulators have been reported to increase BDNF levels in cortical neuron cells through the interaction of BDNF with its receptor tropomyosin-related kinase B (TrKB) and then the activation of Erk1/2 signaling (Fukumoto et al., 2014).

Then, we evaluated the effect of AECs-CM concurrent with Ponesimod and observed they can synergistically promote cell viability. Also, flow cytometry data indicate that hAEC-CM and ponesimod reduced cuprizone-induced apoptosis in oligodendrocytes. In addition, our Western blotting revealed that both treatments hAEC-CM and ponesimod increased the expression of an anti-apoptotic marker of BCL-2 and reduced apoptotic factors including caspase3, caspase8, and Bax. Apoptosis or programmed cell death consists of two pathways: the extrinsic and intrinsic pathways. The intrinsic or mitochondrial pathway happens through the translocation of Bax to the mitochondria, the release of cytochrome c from mitochondria, and protein movement via the mitochondrial membrane which then results in caspases cascade activation including caspase-8 and caspases-3 (Jiao et al., 2012). As shown in the results, the expression of BCL-2 was up-regulated in the OLN-93 cells after treatment with the CM and ponesimod, while the expression of Bax was down-regulated which was led to an increase in the relative BCL-2/Bax ratio. It seems that inhibition of anti-apoptotic protein BCL-2 can be in part a mechanism for cell viability and anti-apoptosis activity of CM and ponesimod. Consistent with our data, in a mouse model of perinatal brain injury, hAEC- CM was effective at reducing injury severity by decreasing the percentage of neural apoptosis (Leaw et al., 2017). Meng et al. and Sankar et al. showed that hAECs can promote the survival of neurons and alter the microenvironment for neural repair (Qiu et al., 2020). Also, the hAEC-CM was found to have neurotrophic effects in a spinal cord injury (Nguyen et al., 2010; Jayapal et al., 2014).

The Western blotting results also demonstrated that ponesimod similar to AECs condition medium increased the expression of anti-apoptotic markers and reduced apoptotic factors caspase3, caspase8, and Bax. Simultaneous administration of hAECs-CM and Ponesimod was an increased anti-apoptotic marker and reduced remarkably apoptotic molecules. Inhibition of apoptosis by ponesimod can be through its main mechanism which is the regulation of sphingosine receptors. Pro-apoptotic sphingosine and ceramide are factors that are balanced with the anti-apoptotic S1P. Sphingosine and ceramide down-regulate the PI3K-Akt pathway in the neural cells which leads to dephosphorylation of Bad and activation of apoptotic pathways at the mitochondria. Also, over-expression of active-Akt neutralizes the apoptotic activity of sphingosine and ceramide (Woodcock, 2006). Also, S1P negatively mediates apoptosis by up-regulating the expression of anti-apoptotic proteins such as BCL-2, and down-regulating the pro-apoptotic protein BAX (Liu et al., 2013).

## Conclusion

We demonstrated here the increasing effects of hAECs-CM and Ponesimod on the viability of the Oligodendrocyte cell line after induction of death by cuprizone in an *in vitro* MS-like model. Data showed that hAEC-CM and Ponesimod act synergistically via inhibition of oligodendrocytes apoptosis. The results suggest that hAECs concurrent with Ponesimod have the potential for use as therapy for MS.

## Data Availability

The raw data supporting the conclusions of this article will be made available by the authors, without undue reservation.
